# Cirsium Setidens Water Extracts Containing Linarin Block Estrogen Deprivation-Induced Bone Loss in Mice

**DOI:** 10.3390/ijms24021620

**Published:** 2023-01-13

**Authors:** Moon-Sik Oh, Soo-Il Kim, Young Eun Sim, Sin-Hye Park, Min-Kyung Kang, Il-Jun Kang, Soon Sung Lim, Young-Hee Kang

**Affiliations:** 1 Department of Food Science and Nutrition and Korean Institute of Nutrition, Hallym University, Chuncheon 24252, Republic of Korea; 2 Department of Food and Nutrition, Andong National University, Andong 36729, Republic of Korea

**Keywords:** acacetin, Cirsium setidens, linarin, ovariectomy, postmenopausal osteoporosis

## Abstract

Osteoporosis is evident in postmenopausal women and is an osteolytic disease characterized by bone loss that further increases the susceptibility to bone fractures and frailty. The use of complementary therapies to alleviate postmenopausal osteoporosis is fairly widespread among women. Edible Cirsium setidens contains various polyphenols of linarin, pectolinarin, and apigenin with antioxidant and hepatoprotective effects. This study aimed to determine whether Cirsium setidens water extracts (CSEs), the component linarin, and its aglycone acacetin blocked ovariectomy (OVX)-induced bone loss. This study employed OVX C57BL/6 female mice as a model for postmenopausal osteoporosis. CSEs, acacetin, or linarin was orally administrated to OVX mice at a dose of 20 mg/kg for 8 weeks. Surgical estrogen loss in mice for 8 weeks reduced bone mineral density (BMD) of mouse femur and serum 17β-estradiol level and enhanced the serum receptor activator of NF-κB ligand/osteoprotegerin ratio with uterine atrophy. CSEs and linarin reversed such adverse effects and enhanced femoral BMD in OVX mice. Oral administration of CSEs and linarin attenuated tartrate-resistant acid phosphate activity and the induction of αvβ3 integrins and proton suppliers in resorption lacunae in femoral bone tissue of OVX mice. In addition, CSEs and linarin curtailed the bone levels of cathepsin K and matrix metalloproteinase-9 responsible for osteoclastic bone resorption. On the other hand, CSEs and linarin enhanced the formation of trabecular bones in estrogen-deficient femur with increased induction of osteocalcin and osteopontin. Further, treatment with CSEs and linarin enhanced the collagen formation-responsive propeptide levels in the circulation along with the increase in the tissue non-specific alkaline phosphatase level in bone exposed to OVX. Supplementing CSEs, acacetin, or linarin to OVX mice elevated the formation of collagen fibers in OVX trabecular bone, evidenced using Picrosirius red staining. Accordingly, CSEs and linarin were effective in retarding osteoclastic bone resorption and promoting osteoblastic bone matrix mineralization under OVX conditions. Therefore, linarin, which is abundant in CSEs, may be a natural compound for targeting postmenopausal osteoporosis and pathological osteoresorptive disorders.

## 1. Introduction

Osteoporosis is characterized by microarchitectural deterioration of bone tissue leading to increased bone fragility and fractures [1,2]. In postmenopausal women, estrogen deprivation causes high bone turnover and loss, leading to primary osteoporosis [3,4]. The reduction in the estrogen level at menopause is one of the strong risk factors for developing postmenopausal osteoporosis [5]. Thus, the use of estrogen therapy is fairly widespread among menopause-experiencing women in order to effectively treat adverse bone malformations and cardiovascular diseases [6,7]. However, long-term hormone therapy may increase the risk of breast cancer and other chronic diseases [6]. Accordingly, the increased risk of long-term hormone therapy triggers the use of harmless and affordable alternative therapies [8]. In addition, several pharmacological medications potentially improving bone strength help the recovery of bone mass in postmenopausal osteoporosis [4,7,9]. Such medications include calcium, vitamin D, bisphosphonates, and denosumab [2,10]. In fact, the osteoporotic risks of menopause women can be alleviated through changes in lifestyle in terms of multiple types of diet and exercise [11].

Numerous studies have reported that nonsteroidal compounds have beneficial effects on postmenopausal bone health [8,12]. Phytoestrogens, as nonsteroidal plant compounds, have structural similarity with 17*β*-estradiol primarily through binding to estrogen receptors [13,14]. Isoflavones are a type of naturally occurring isoflavonoids and phytoestrogens with estrogen-like biological activity [15,16]. A growing body of evidence demonstrates that phytoestrogens, including prenylated flavonoids, coumestans, and lignans, as well as isoflavones, have biological potency on bone in mammals as naturally occurring anti-osteoporotic agents [17]. These compounds inhibit osteoclastic osteoporosis via multiple targets and specific pathways [18,19,20]. For instance, the isoflavone daidzein prevents bone loss by reversing detrimental immune changes [21]. In addition, resveratrol displays bone-protective effects by antagonizing adipogenesis [22]. Furthermore, this compound promotes the osteogenesis of human mesenchymal stem cells via the SIRT1/FOXO3A axis as a novel mechanism [23].

Especially, phytoestrogens combat bone resorption provoked by estrogen deficiency by suppressing osteoclastic activation [8,12,17]. The isoflavone formononetin has favorable effects on the mechanical properties and chemical composition of bone in ovariectomized (OVX) rats [24]. Our previous study has shown that the apple polyphenol phloretin inhibits OVX-induced osteoclast differentiation-associated osteoporosis in mice [25]. Oral administration of milk thistle (Silybum marianum) extracts prevents OVX-induced bone loss by blocking osteoclast differentiation and activation [26]. However, their action mechanisms for manipulating bone-specific remodeling remain unclear under the OVX condition. On the other hand, little evidence is available to support osteoblastogenesis of phytoestrogens in estrogen deprivation-induced postmenopausal osteoporosis.

With the growing availability of phytoestrogens, the current study attempted to examine whether Cirsium setidens water extracts (CSEs) inhibited estrogens deficiency-associated osteoporosis. This study evaluated the effects of CSEs on the histological status of femoral bone and the tissue levels of bone-specific proteins in OVX mice. Additionally, the inhibitory effects of linarin and its aglycone acacetin on primary osteoporosis were determined in OVX mice. It has been previously shown that linarin and pectolinarin are identified as major components of CSEs [27]. OVX mice were daily fed with a diet containing 20 mg/kg CSEs, 20 mg/kg acacetin, or 20 mg/kg linarin for 8 weeks. In contrast, sham-operated mice were fed with the control diet. This study found that CSEs attenuated bone loss induced by estrogen deficiency by inhibiting osteoclastogenesis and promoting osteoblastogenesis and bone matrix formation. Therefore, CSEs containing linarin could be an alternative treatment for the prevention of postmenopausal osteoporosis.

## 2. Results

### 2.1. Effects of CSEs on OVX-Induced Uterine Atrophy

**Figure 1:**
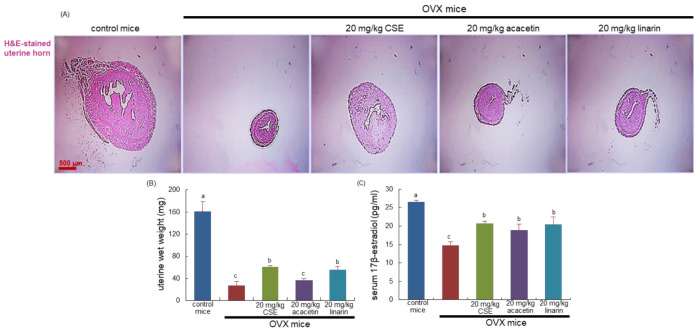
Histological staining of uterus transverse section (**A**), wet weight of uterine tissues (**B**), and serum 17β-estradiol level (**C**) of ovariectomized (OVX) mice orally treated with 20 mg/kg Cirsium setidens water extracts (CSEs), acacetin, or linarin daily for 8 weeks. Cross-sectional images of the uterine horn were obtained by staining with hematoxylin and eosin (H&E) and were visualized using light microscopy. Scale bar = 500 μm. The serum 17β-estradiol level was determined using an enzyme-linked immunosorbent assay kit. Values in bar graphs (mean ± SEM, *n* = 9) not sharing a small alphabetical letter are different at *p* < 0.05.

OVX-induced 8-week estrogen loss resulted in a marked atrophy of the uterus (Figure 1A). Such significant reduction was observed in the size and wet weight of the uterus due to OVX (Figure 1B). Administration of 20 mg/kg CSEs to OVX mice improved uterus size and wet weight. In addition, 20 mg/kg linarin but not 20 mg/kg acacetin increased both the size and wet weight of OVX mouse uterus (Figure 1C). Further, the serum level of 17β-estradiol declined in OVX mice and was significantly blocked by the administration of all the CSEs, acacetin, and linarin (Figure 1D).

### 2.2. Elevation of Osteoclastic Activation Induced by CSEs in OVX Mice

**Figure 2:**
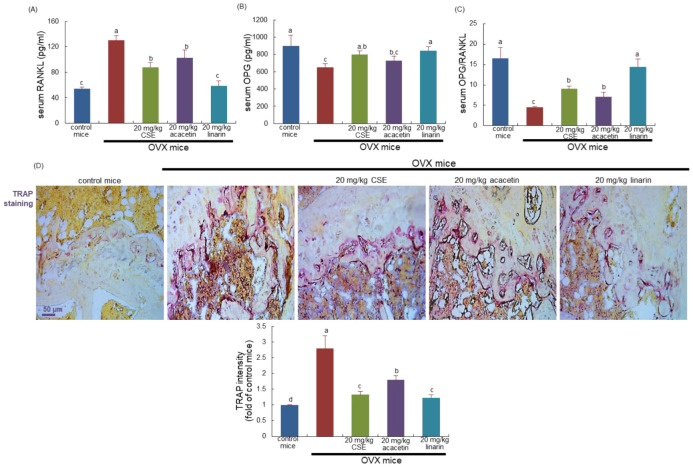
Circulating levels of receptor activator of nuclear factor kappa-Β ligand (RANKL; **A**) and osteoprotegerin (OPG; **B**), OPG/RANK ratio (**C**), and tartrate-resistant acid phosphatase (TRAP) localization and activity (**D**) in ovariectomized (OVX) mice. OVX mice were orally supplemented with 20 mg/kg Cirsium setidens water extracts (CSEs), acacetin, or linarin daily for 8 weeks. The serum levels of RANKL and OPG were measured by employing enzyme-linked immunosorbent assay kits. Values in bar graphs (mean ± SEM, *n* = 4) not sharing a small alphabetical letter are different at *p* < 0.05. Longitudinal femoral bone tissues were stained with TRAP staining kits, and absorbance for TRAP intensity (mean ± SEM, *n* = 4) was measured at λ = 405 nm. Scale bar = 50 μm.

This study examined the serum levels of receptor activator of nuclear factor-κB ligand (RANKL) and osteoprotegerin (OPG) at the 8th week following surgical ovary deprivation. The serum RANKL level was highly elevated due to uterine loss, and conversely, the serum OPG level declined significantly in OVX mice, as compared with that of sham-operated mice (Figure 2A,B). When 20 mg/kg CSEs or 20 mg/kg linarin was supplemented for 8 weeks after OVX, the serum levels of RANKL and OPG were reversed, thus enhancing the serum OPG/RANKL ratio (Figure 2C). However, 20 mg/kg acacetin did not have such favorable effect on the serum OPG/RANKL ratio. Accordingly, CSEs and linarin may hamper osteoclastogenesis in the osteoporosis-prone bone of OVX mice.

### 2.3. Effects of CSEs on Tartrate-Resistant Acid Phosphate (TRAP) Activity and Bone Mineral Density (BMD) in OVX Mouse Bone

This study attempted to confirm that CSEs inhibited osteoclast differentiation in the bone of OVX mice, evidenced using TRAP staining. At 8 weeks after OVX, there were many purple-stained TRAP-positive osteoclasts detected in the femur of OVX mice (Figure 2D). On the contrary, the dark purple staining became weak in the femoral bone of OVX mice treated with 20 mg/kg CSEs (Figure 2D). Furthermore, the treatment of linarin and acacetin inhibited the osteoclastic TRAP activity in the femoral bone of OVX mice (Figure 2D). Accordingly, linarin and acacetin as well as CSEs may reduce osteoclastic bone resorption induced by OVX.

BMD and bone mineral content (BMC) in mouse femur and tibia were significantly diminished 8 weeks after OVX (Table 1). However, the supplementation of OVX mice with 20 mg/kg CSEs and 20 mg/kg linarin substantially elevated the femoral and tibial BMD and BMC in mice, as compared with sham-operated control mice (Table 1). When estrogen-deficient mice were treated with 20 mg/kg acacetin, the increments in BMD and BMC were minimal (Table 1).

### 2.4. Inhibitory Effects of CSEs on Acidification of the Resorption Lacunae

**Figure 3:**
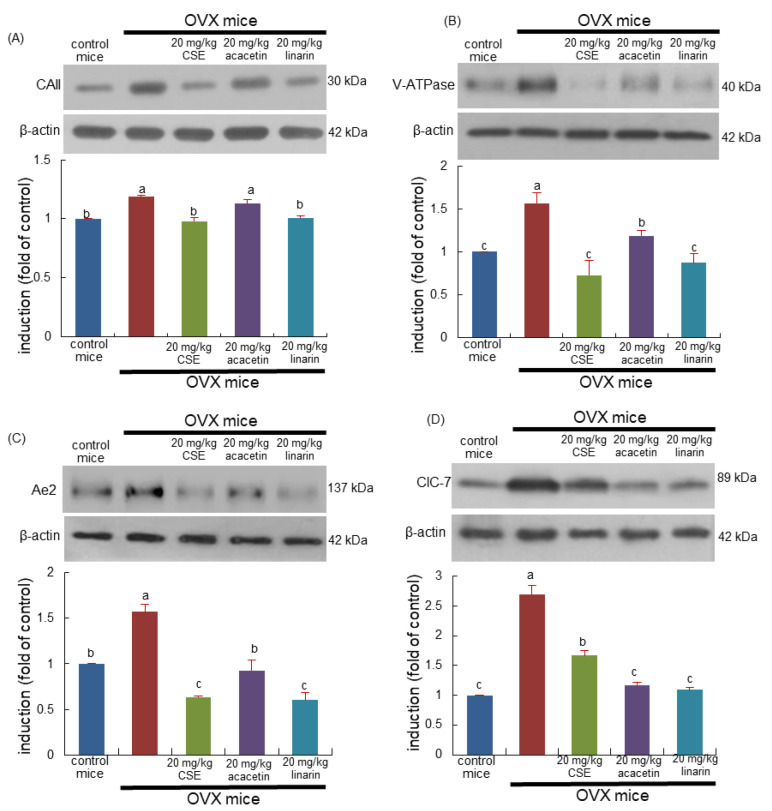
Inhibition of induction of osteoclastic proteins of ovariectomized (OVX) mice induced by Cirsium setidens water extracts (CSEs). OVX mice were orally supplemented with 20 mg/kg CSEs, 20 mg/kg acacetin, or 20 mg/kg linarin daily for 8 weeks. Whole bone tissue extracts were subjected to SDS-PAGE and Western blot analyses with specific antibodies against carbonic anhydrase II (CAII; **A**), vacuolar ATPase (V-ATPase; **B**), anion exchanger 2 (Ae2; **C**), and chloride channel (ClC)-7 (**D**). β-Actin was used as internal control. The bar graphs (mean ± SEM, *n* = 3) represent quantitative results of bands obtained with a densitometer. Values in bar graphs not sharing a small alphabetical letter at *p* < 0.05.

This study examined whether CSEs inhibited the induction of osteoclastogenic markers related to the proton formation and translocation in estrogen-deficient mice. Western blot data revealed that the ovary loss induced carbonic anhydrase II (CAII) and vacuolar-type H(+)- ATPase (V-ATPase) in bone as proton suppliers for bone resorption (Figure 3A,B). Treatment with CSEs or linarin attenuated the induction of osteoclastic CAII and V-ATPase in OVX mouse bone. However, linarin aglycon acacetin was ineffective in inhibiting the induction of the proton suppliers in the bone resorption lacunae (Figure 3A,B). Accordingly, CSEs and linarin may block bone resorption through the acidification of the resorption lacunae in the osteoclast–bone interface.

The tissue level of Cl-/HCO3- exchanger Ae2 was enhanced in OVX mouse bone and was inhibited by treating mice with 20 mg/kg CSEs (Figure 3C). In addition, CSEs reduced the tissue level of Cl- ion channel chloride channel 7 (CIC-7) augmented by OVX (Figure 3D). Similarly, linarin inhibited the induction of Ae2 and ClC-7 as cellular mechanism for Cl- during bone resorption. Thus, CSEs and linarin deterred the creation of the acidic microenvironment for resorption in the bone of estrogen-deficient mice.

### 2.5. Inhibition of Sealing Zone Formation and Lysosomal Enzyme Secretion Induced by CSEs

**Figure 4:**
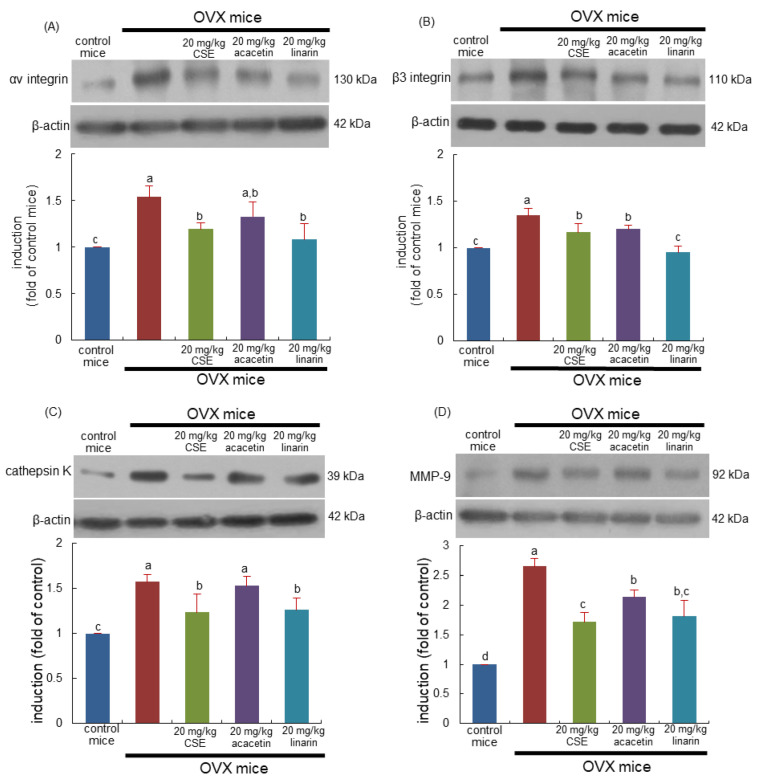
Blockade of induction of αv integrin (**A**), β3 integrinin (**B**), cathepsin K (**C**), and matrix metalloproteinase (MMP)-9 (**D**) induced by Cirsium setidens water extracts (CSEs) in femoral bone tissue sections of ovariectomized (OVX) mice. OVX mice were orally supplemented with 20 mg/kg CSEs, acacetin, or linarin daily for 8 weeks. Western blot data of bone tissues were obtained using anti-αv integrin, anti-β3 integrinin, anti-cathepsin K, and anti-MMP-9, and β-actin protein was used as internal control. The bar graphs (mean ± SEM) in the right panels represent quantitative results obtained using a densitometer. Values in bar graphs not sharing a small alphabetical letter are different at *p* < 0.05.

This study determined whether CSEs suppressed the induction of αvβ3 integrin localized in the sealing zone of osteoclasts. The induction of both αv integrin and β3 integrin in bone tissue was elevated in OVX mice (Figure 4A,B). In contrast, 20 mg/kg CSEs and 20 mg/kg linarin substantially diminished the induction of αvβ3 integrin proteins. In addition, acacetin inhibited the induction of β3 integrin but not αv integrin (Figure 4A,B). Consequently, CSEs containing linarin may suppress the formation of the sealing zone required for osteoclastic bone resorption activated by OVX.

This study further examined whether CSEs attenuated osteoporotic bone resorption owing to ovary loss. OVX enhanced the induction of lysosomal cysteine protease cathepsin K (Figure 4C). In addition, the collagen-degrading matrix metalloproteinase-9 (MMP-9) level was enhanced in OVX mouse bone (Figure 4D). However, the administration of 20 mg/kg CSEs or 20 mg/kg linarin to OVX mice reduced the induction of both lytic enzymes. Acacetin treatment attenuated the MMP-9 induction enhanced by OVX (Figure 4D). Collectively, CSEs may retard the degradation of bone organic matrix by inhibiting osteoclastic proteases, including cathepsin K and MMP-9.

### 2.6. Formation of Trabecular Bone Induced by CSEs in OVX Mice

**Figure 5:**
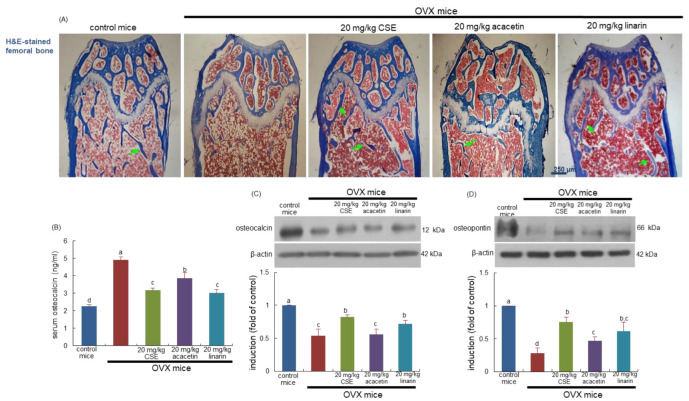
Effects of Cirsium setidens water extracts (CSEs), acacetin, or linarin on histology of trabecular bones (**A**), serum level of osteocalcin (**B**), and bone tissue levels of osteocalcin (**C**) and osteopontin (**D**) in ovariectomized (OVX) mice. OVX mice were orally supplemented with 20 mg/kg CSEs, 20 mg/kg acacetin, or 20 mg/kg linarin daily for 8 weeks. For the observation of trabecular bone and epiphyseal plate (**A**), longitudinal sections of femoral bone tissue were hematoxylin and eosin (H&E)-stained. Images were visualized using light microscopy (four separate experiments). Green arrows indicate the formation of trabecular bones. Scale bar = 250 μm. The serum level of osteocalcin was measured by employing an enzyme-linked immunosorbent assay kit (**B**). Whole bone tissue extracts were subjected to SDS-PAGE and Western blot analyses with specific antibodies against osteocalcin (**C**) and osteopontin (**D**). β-Actin was used as internal control. The bar graphs (mean ± SEM) in the right panels represent quantitative results obtained using a densitometer. Values in bar graphs not sharing a small alphabetical letter are different at *p* < 0.05.

For 8 weeks post OVX, bony meshwork was observed in the femoral diaphysis and metaphysis of OVX mice (Figure 5A). When OVX mice were treated with CSEs, the trabecular bones were newly formed in the metaphysis and diaphysis (green arrows). Treatment with linarin and acacetin histologically improved the sagittal regions of the femur (Figure 5A). Oral administration of 20 mg/kg acacetin appeared to enhance the formation of trabecular bones in the femur of OVX mice. Accordingly, CSEs with linarin may be effective in newly forming trabecular bones gone away during ovary loss.

This study investigated the induction of osteoblastic markers of osteocalcin and osteopontin in OVX mice treated with CSEs. Ovary loss highly increased the serum level of osteocalcin in mice, while its bone level was reduced (Figure 5B,C). When OVX mice were orally administrated with CSEs, such effects were reversed. Additionally, osteopontin induction declined in the bone of OVX mice and was blocked by supplying 20 mg/kg CSEs to mice (Figure 5D). In mice treated with 20 mg/kg linarin, similar effects on the bone tissue levels of osteocalcin and osteopontin were observed (Figure 5C,D). Accordingly, the induction of both non-collagenous proteins of osteocalcin and osteopontin by CSEs may be responsible for the formation of new trabecular bones.

### 2.7. Effects of CSEs on Collagen Metabolism

**Figure 6:**
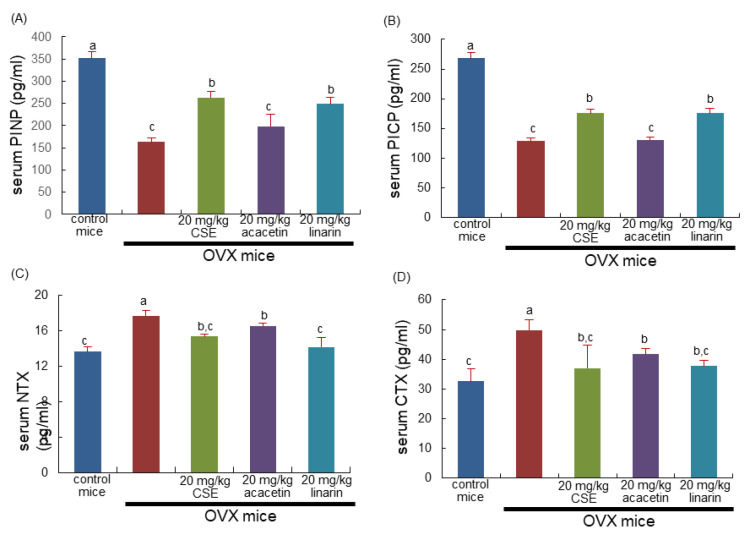
Serum levels of collagen formation and degradation in ovariectomized (OVX) mice treated with Cirsium setidens water extracts (CSEs). OVX mice were orally supplemented with 20 mg/kg CSEs, acacetin, or linarin daily for 8 weeks. The serum levels of procollagen type 1 amino-terminal propeptide (PINP; **A**), procollagen type 1 carboxy-terminal propeptide (PICP; **B**), amino-terminal telopeptide of type 1 collagen (NTX-1; **C**), and carboxy-terminal telopeptide of type 1 collagen (CTX-1; **D**) were measured using ELISA kits. Respective values (mean ± SEM, *n* = 3) in bar graphs not sharing an alphabetical lowercase letter (a, b, c) are different at *p* < 0.05.

The current study investigated the beneficial effects of CSEs on collagen formation in OVX mice. The serum levels of collagen synthesis-associated procollagen type 1 amino-terminal propeptide (PINP) and procollagen type 1 carboxy-terminal propeptide (PICP) were reduced in OVX mice, as evidenced using ELISA (Figure 6A,B). Treatment with CSEs and linarin significantly elevated the serum levels of these propeptides lessened by OVX. It was further examined whether CSEs influenced the serum levels of amino-terminal telopeptide of type 1 collagen (NTX-1) and carboxy-terminal telopeptide of type 1 collagen (CTX-1), both released during collagen degradation. The release of NTX-1 and CTX-1 into the circulation was highly promoted in OVX mice (Figure 6C,D). The increased release of both collagen-related biomarkers was reduced by the treatments with CSEs, acacetin, or linarin. Thus, CSEs were effective in optimally maintaining the collagen-mediated bone matrix deteriorated by estrogen deprivation.

### 2.8. Effects of CSEs on Mineralization of Collagen Bone Matrix

**Figure 7:**
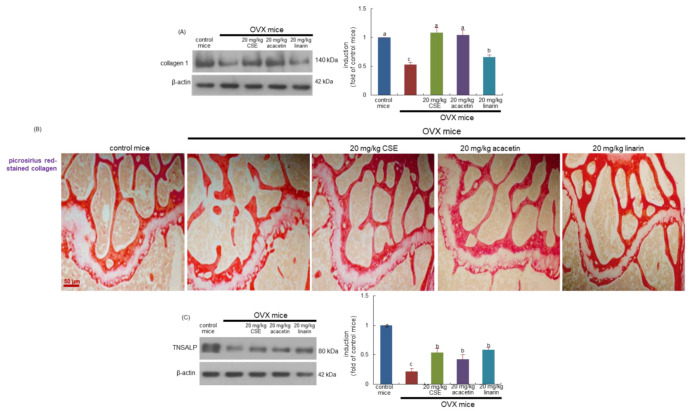
Boosting effects of Cirsium setidens water extracts (CSEs), acacetin, or linarin on induction of collagen type-1 (**A**), formation of trabecular collagen fibers (**B**), and tissue level of tissue non-specific alkaline phosphatase (TNSALP; C) in femoral bone of ovariectomized (OVX) mice. OVX mice were orally supplemented with 20 mg/kg CSEs, 20 mg/kg acacetin, or 20 mg/kg linarin daily for 8 weeks. Whole bone tissue extracts were subjected to SDS-PAGE and Western blot analyses with specific antibodies against collagen type 1 and TNSALP (**A**,**C**). β-Actin was used as internal control. The bar graphs (mean ± SEM) in the right panels represent quantitative results obtained using a densitometer. Values in bar graphs not sharing a small alphabetical letter are different at *p* < 0.05. Picrosirius red staining was conducted for detecting trabecular bone collagen (**B**). The black arrows indicate the depletion of collagen fibers in trabecular bones of the epiphysis. Representative images were visualized using light microscopy. Scale bar = 50 μm.

This study found that the bone level of collagen type 1 was diminished in OVX mice, as compared with sham-operated mice (Figure 7A). When OVX mice were treated with CSEs and acacetin, the bone tissue level of collagen 1 was restored to that of control mice (Figure 7A). In addition, linarin was effective in increasing the collagen 1 level. Accordingly, CSEs with linarin may promote the formation of collagenous bone matrix. Picrosirius red staining revealed that whitish patchy marks could be observed in the femoral bone tissue of OVX mice, indicating that collagen fibers were diminished in femoral bone (Figure 7B). However, the oral administration of CSEs or linarin enhanced collagen fibers in the femur of OVX mice. Accordingly, it can be assumed that CSEs restored the collagen mineralization and formation of collagenous bone matrix that failed due to OVX.

This study further explored whether CSEs promoted bone matrix mineralization in OVX-challenged bone. Estrogen loss reduced the induction of the tissue non-specific alkaline phosphatase (TNSALP) enzyme catalyzing inorganic pyrophosphate to phosphate on the plasma membrane and matrix vesicles of osteoblasts (Figure 7C). In contrast, CSEs and linarin increased the bone tissue level of TNSALP in OVX mice. Thus, CSEs may lead bone matrix mineralization involving calcium ions and phosphate ions.

## 3. Discussion

The nine major findings were obtained from this study by employing CSEs containing linarin: (1) The administration of 20 mg/kg CSEs, 20 mg/kg acacetin, and 20 mg/kg linarin to OVX mice did not induce liver toxicity and improved the blood lipid profile. (2) CSEs and linarin inhibited uterine atrophy in OVX mice without a marked reduction in serum 17β-estradiol. (3) Treatment with CSEs and linarin elevated the serum OPG/RANKL ratio and decreased femoral TRAP activity in OVX mice, along with increases in femoral and tibial BMD and BMC. (4) Treatment with CSEs and linarin attenuated the induction of osteoclastogenic markers of CAII, V-ATPase, Ae2, and ClC-7 in OVX mouse bone. (5) CSEs with linarin substantially reduced the bone tissue levels of αvβ3 integrin proteins, MMP-9, and cathepsin K. (6) Treatment of OVX mice with CSEs, linarin, and acacetin enhanced the formation of trabecular bones in the femur. (7) CSEs and linarin enhanced the induction of osteocalcin and osteopontin in estrogen-deficient bone. (8) Treatment with CSEs, acacetin, and linarin favorably influenced the serum levels of propeptides responsible for collagen formation and degradation, leading to increased formation of collagen fibers in OVX bone. (9) CSEs and linarin increased the bone tissue level of TNSALP in OVX mice. Therefore, CSEs containing linarin may attenuate osteoclastogenic bone resorption and boost osteogenesis and collagen-mediated bone matrix mineralization in OVX-induced osteoporotic bone.

In postmenopausal osteoporosis, which results from estrogen deficiency, the imbalance in bone formation and resorption has adverse effects on trabecular and cortical bones, leading to trabecular connectivity loss and cortical thinning and porosity [3,4,5]. Several drugs, such as bisphosphonates, denosumab, and teriparatide, are known to potentially reduce fracture risk and improve bone strength by slowing down bone resorption or by stimulating bone formation [7,9,10]. The mechanistic understanding of the cellular basis for estrogen deprivation-induced osteoporosis has allowed researchers to develop new pharmacological medications targeted to key pathways. In addition, the preventive strategies to improve bone health in postmenopausal osteoporosis include multiple changes in lifestyle and the use of estrogen therapy [11]. However, long-term estrogen therapy may result in increased risk of breast cancer and unfavorable net effects on inflammatory and immune factors [6]. Consequently, there is a need for the use of promising alternative therapies [8,12,15]. A growing body of evidence supports that nonsteroidal compounds have favorable effects on bone health via exerting estrogen-like biological activity [13,14,16,17].

Phytoestrogens such as isoflavones and prenylated flavonoids prevent resorptive bone diseases caused by estrogen deficiency through inhibiting osteoclastic activation [13,14,19]. These compounds are known as naturally occurring anti-osteoporotic agents that mainly inhibit osteoclastogenesis via multiple targets and specific pathways [21,22,23]. So far, the action mechanisms of these compounds are emerging in regard to osteoclastogenesis under estrogen-deficient conditions. The current study revealed that oral administration of CSEs and linarin improved femoral and tibial BMD in OVX mice and increased the level of serum 17β-estradiol. In addition, treatment with CSEs attenuated osteoclastogenesis in the bone of OVX mice. The current product diminished the tissue levels of osteoclast-specific markers responsible for the formation of giant osteoclasts with actin rings and for the degradation of bone organic matrix at the osteoclast–bone interface. In fact, linarin and acacetin attenuated the focal adhesion of osteoclasts to bone matrix via the inhibition of αvβ3 integrin and CD44 [27]. In our previous study, the flavones of linarin and pectolinarin were identified as major components present in CSEs [27]. Linarin but not pectolinarin was effective in hampering the focal adhesion of cultured osteoclasts to bone matrix and in curtaining active bone resorption. Thus, it can be speculated that such mechanistic action of linarin could be responsible for the favorable effect of CSEs on postmenopausal osteoporosis. The antiosteoporotic effects of CSEs are thought to have resulted from other phenolic compounds, as well as linarin, present in CSEs. Linarin aglycon acacetin partially blocked the induction of osteoclast-specific marker proteins. Accordingly, the glucose moiety of linarin appeared to be involved in the inhibition of osteoclastogenesis.

Several studies have shown that natural plant compounds promote osteoblastic bone formation via regulating multiple signaling pathways, e.g., AKT/mTOR, BMP-2/Smad, and Wnt/β-catenin [28,29,30]. However, little evidence is available to support the osteoblastogenesis of phytoestrogens in postmenopausal osteoporosis. This study found that oral treatment with CSEs or linarin enhanced osteoblast differentiation and bone matrix formation in estrogen-deficient mice, leading to the increase in femoral and tibial BMD. Since collagen type 1 plays a role in controlling bone strength [31,32], CSEs and linarin can be said to have increased bone strength in osteoporotic bone. In addition, linarin, which is abundant in CSEs, may act as a phytoestrogen, improving uterine atrophy and boosting osteoblastogenesis under estrogen deficiency. Unfortunately, this study did not examine the mechanistic signaling pathway(s) responsible for the osteogenic activity of CSEs and linarin. A clinical trial reports that icariin, a prenylated flavonol glycoside, is effective in preventing postmenopausal osteoporosis with bone-promoting activity and relatively low side effects [33,34]. Thus, high-quality clinical studies on CSEs are necessary to evidence beneficial and safe antiosteoporotic application in menopausal women.

## 4. Materials and Methods

### 4.1. Materials

Acacetin and linarin were purchased from Sigma-Aldrich Chemical (St. Louis, MO, USA), as were all other reagents, unless specifically stated elsewhere. Antibodies of mouse CAII and ClC-7 were provided by Abcam Biochemicals (Cambridge, UK). Antibodies of mouse V-ATPase, mouse MMP-9, mouse cathepsin K, mouse integrin αv, mouse osteocalcin, mouse osteopontin, mouse TNSALP, and mouse collagen type I were obtained from Santa Cruz Biotechnology (Santa Cruz, CA, USA). Mouse integrin β3 antibody was purchased from Cell Signaling Technology (Beverly, MA, USA). Horseradish peroxidase (HRP)-conjugated goat anti-rabbit IgG, donkey anti-goat IgG, and goat anti-mouse IgG were supplied by Jackson ImmunoResearch Laboratory (West Grove, PA, USA). Mouse monoclonal β-actin antibody was purchased from Sigma-Aldrich Chemical (St. Louis, MO, USA).

### 4.2. Preparation of CSEs

The plant Cirsium setidens was provided by Jeongseon-Gondre Farming Association Corporation (Jeongseon-gun, Republic of Korea). Extraction of dried leaves of Cirsium setidens (100 g) was performed with a Soxhlet extractor (MS-EAM; Misung, Yangju, Republic of Korea) by suspending them in hot distilled water (1 L, 80 °C) for 8 h. After reflux, the crude CSEs were centrifuged at 5000 rpm for 30 min and filtered to remove plant particles with Watman No.1 filter paper. The filtered extracts were then concentrated and lyophilized using a freeze-dryer in order to fully dry the extracts for use [27].

### 4.3. Animals and Ovariectomy

To investigate the inhibitory effects of CSEs, acacetin, and linarin on osteoporotic activity in an estrogen-deficient animal model, this study introduced the ovariectomical technique mimicking estrogen deprivation or senescent menopause. C57BL/6 mice (11 weeks of age, 20–25 g) were obtained from DBL (Eumsung, Republic of Korea) and kept on a 12 h light/dark cycle at 20–25 °C with 60% relative humidity under specific pathogen-free conditions. Mice were fed a non-purified diet (RodFeed, DBL, Eumsung, Republic of Korea) during the 8-week experimental period with free access to water ad libitum at the animal facility of Hallym University. The animals were allowed to acclimatize for a week before experiments were commenced. All the animal experiments were performed in accordance with the university’s Guidelines for the Care and Use of Laboratory Animals approved by Committee on Animal Experimentation of Hallym University (permission number: Hallym 2019-31).

For the ovariectomical surgery [25,26], 11-week-old female animals were anesthetized using a ketamine/Rompun cocktail (40 mg ketamine/kg and 10 mg rompun/kg body weight) for either a sham operation (Sham) or bilateral oophorectomy. Mice receiving surgical OVX were orally treated with 20 mg/kg CSEs, 20 mg/kg acacetin, or 20 mg/kg linarin once a day for 8 weeks (9–12 mice in each group). After 8 weeks of treatment, blood samples and uterine tissues were collected, and serum samples obtained by means of centrifugation (3000 rpm, 10 min) were stored at −70 °C prior to biochemical and enzyme-linked immunosorbent assay (ELISA) analyses.

Eight weeks following ovariectomical surgery, the final body weight (BW) and average daily gain (ADG) significantly increased in OVX mice, as compared with sham-operated control mice (Table 2). A decreasing tendency was observed in the final BW and ADG of OVX mice supplemented with CSEs, acacetin, and linarin. No significant differences were observed in the average daily feed intake (ADFI) of each mouse group. However, the food efficiency ratio (FER = ADG/ADFI) was significantly higher than that of the other groups (Table 2). Ovariectomy reduced the liver-weight-to-BW ratio (LW/BW) as compared with sham-operated control mice.

### 4.4. Biochemical Analyses in Serum and Bone

Serum levels of glutamic pyruvic transaminase (GPT) and glutamic oxaloacetic transaminase (GOT) were determined after overnight fasting using Kornelab 20XT (Thermo Fisher Scientific Inc., Waltham, MA, USA). This study examined the serum levels of total cholesterol (TC), triglyceride (TG), high-density lipoprotein cholesterol (HDL-C), and low-density lipoprotein cholesterol (LDL-C) using an automated clinical chemistry analyzer (DRI-CHEM NX500i; FUJIFILM Corp., Tokyo, Japan) according to the manufacturer’s instructions. The serum levels of GOT and GPT were not influenced by the administration of CSEs, acacetin, or linarin to OVX mice, indicating that they did not induce liver toxicity (Table 2). In addition, the levels TC, TG, and LDL-C significantly increased after OVX surgery, whereas the HDL-C level declined (Table 3). The blood lipid profiles were improved in OVX mice treated with CSEs, acacetin, and linarin.

The BMD and BMC in mouse femoral and tibial bones were measured with a PIXImus mouse densitometer (GE Lunar, Waukesha, WI, USA). BMD, calculated with BMC (mg) and the projected bone area (cm^2^), was determined in the femoral and tibial regions.

### 4.5. Histological Examination of Uterus and Bone

Uterus and femoral bone tissues were obtained from three mice of each group. After being washed with saline, uterine tissues were fixed in 10% neutral buffered formalin for 24 h. Uterine tissues were stained using a modified Harris hematoxylin and Shandon instant eosin (H&E) for microscopic observation. Paraffin-embedded femoral bone tissues cut into cryostat sections 5 µm in thickness were deparaffinized and hydrated with xylene and graded ethanol. H&E staining was applied for the histological observation of bone tissues.

After each slide with tissue sections was mounted in VectaMount mounting medium (Vector Laboratories, Burlingame, CA, USA), images were taken using an Axiomager optical microscope system (Carl Zeiss, Jena, Germany).

### 4.6. TRAP Staining

For the histological staining of TRAP-positive osteoclasts, femoral bone tissues embedded in paraffin were cut into sections 5 μm in thickness. TRAP staining was conducted using a leukocyte acid phosphatase kit (Sigma-Aldrich Chemicals, St. Louis, MO, USA). The femoral bone samples were incubated for 20 min in 50 mM sodium acetate and 40 mM potassium sodium tartrate buffer (pH 5.0) and further incubated for 15 min in the same buffer containing 2.5 mg/mL Naphthol AS-BI phosphate and 0.5 mg/mL Fast Garnet GBC. Images of tissue sections on slides were taken using an Axiomager optical microscope system. For the measurement of TRAP activity, absorbance for TRAP intensity was measured at λ = 405 nm.

### 4.7. Western Blot Analysis

The bone fragments were trimmed, washed to remove soft tissues and contaminants with PBS, and incubated at 4 °C overnight in 1.2 M HCl to demineralize the bone tissues, as described in our previous studies [25,26]. Equal amounts of extract proteins were electrophoresed on 6–12% SDS-PAGE gels and transferred onto a nitrocellulose membrane. Non-specific binding was blocked by soaking membranes in TBS-T buffer (50 mM Tris-HCl (pH 7.5), 150 mM NaCl, and 0.1% Tween20) containing 3% bovine serum albumin or 5% non-fat milk for 3 h. The membranes were incubated with the primary antibody of the target proteins. The membranes were then incubated with goat anti-rabbit IgG conjugated to horseradish peroxidase as a secondary antibody. The protein levels on gels were determined using Supersignal West Pico Chemiluminescence detection reagents (Pierce Biotechnology, Rockford, IL, USA) and Konica X-ray film (Konica, Tokyo, Japan). Incubation with monoclonal mouse antibody of β-actin was conducted as a comparative control.

### 4.8. Enzyme-Linked Immunosorbent Assay (ELISA)

The serum 17β-estradiol level was determined using ELISA kits (R&D Systems, Minneapolis, MN, USA) according to the manufacturer’s instructions [35]. The serum levels of OPG and RANKL were also determined using ELISA assay kits (R&D Systems, Minneapolis, MN, USA). In addition, the serum levels of PICP, PINP, CTX-1, and NTX-1 were measured using ELISA kits (Novus Biologicals, Centennial, CO, USA) according to the manufacturer’s instructions [36]. The serum osteocalcin level was also measured using an ELISA kit (Life Technologies, Carlsbad, CA, USA).

### 4.9. Collagen Fiber Staining of Bones

Picrosirius red staining was employed for detecting bone collagen fibers. Paraffin-embedded femoral bone tissues cut into cryostat sections 5 µm in thickness were deparaffinized and hydrated with xylene and graded ethanol. The tissue sections were incubated with Picrosirius red solution (Sigma-Aldrich chemical, St. Louis, MO, USA) overnight at room temperature. After each slide was mounted, images were taken using an optical Axiomager microscope system.

### 4.10. Statistical Analyses

Statistical analyses of experimental data (mean ± SEM) were performed using Statistical Analysis Systems statistical software package (SAS Institute, Cary, NC, USA). Significance was determined using one-way analysis of variance, followed by Duncan’s range test for multiple comparisons. Differences were considered significant at *p* < 0.05.

## 5. Conclusions

The current report demonstrates that CSEs and linarin inhibited osteoclast activity and stimulated osteoblast activity in OVX mice, leading to the suppression of bone loss and the increase in bone strength. The oral administration of CSEs and linarin attenuated the atrophy of the uterus induced by OVX and improved the serum 17β-estradiol level. The administration of CSEs and linarin to OVX mice inhibited the induction of proton suppliers in resorption lacunae, integrins, and bone matrix-degrading enzymes. Further, these products boosted the induction of both collagen 1 and non-collagenous proteins, indicating matrix mineralization in estrogen-deficient bone via regulating collagen formation and degradation. Therefore, CSEs containing linarin may be effective in ameliorating pathological osteoresorptive disorders such as postmenopausal osteoporosis by orchestrating osteogenesis and osteoclastogenesis. However, the favorable and sustainable efficacy of edible Cirsium setidens in the diet needs to be defined for bone health at a clinical level.

## Figures and Tables

**Table 1:** Osteogenic activity of CSEs, acacetin, and linarin in OVX mice.

	Animals	Control Mice	OVX Mice	OVX Mice		
Parameters				20 mg/kg CSE	20 mg/kg Acacetin	20 mg/kg Linarin
Femur	BMD	82.53 ± 0.54 ^a^	71.43 ± 0.41 ^c^	78.77 ± 0.58 ^b^	74.72 ± 0.63 ^b^	78.22 ± 0.59 ^b^
	BMC	18.83 ± 0.48 ^a^	17.14 ± 0.40 ^b^	18.50 ± 0.22 ^a^	17.33 ± 0.33 ^b^	18.33 ± 0.33 ^a^
	area	0.19 ± 0.002 ^a^	0.20 ± 0.003 ^a^	0.20 ± 0.003 ^a^	0.19 ± 0.003 ^a^	0.20 ± 0.005 ^a^
Tibia	BMD	64.07 ± 0.32 ^a^	55.84 ± 0.26 ^c^	61.70 ± 0.31 ^b^	56.98 ± 0.42 ^c^	59.22 ± 0.35 ^b^
	BMC	10.17 ± 0.31 ^a^	9.57 ± 0.20 ^b^	9.83 ± 0.31 ^a,b^	9.50 ± 0.22 ^b^	9.80 ± 0.17 ^a,b^
	area	0.15 ± 0.002 ^a^	0.15 ± 0.003 ^a^	0.15 ± 0.003 ^a^	0.15 ± 0.002 ^a^	0.15 ± 0.004 ^a^

Ovariectomized (OVX) C57BL/6 female mice were orally administrated with 20 mg/kg Cirsium setidens water extracts (CSEs), 20 mg/kg acacetin, and 20 mg/kg linarin daily for 8 weeks. The bone mineral density (BMD; mg/cm^2^), bone mineral content (BMC; mg), and bone area (cm^2^) of mouse tissues of femur and tibia were determined using a PIXImus mouse densitometer. Respective values in the same column (mean ± SEM, *n* = 10) not sharing a small alphabetical letter are different at *p* < 0.05.

**Table 2:** Changes in body weight gain, feed intake, and other parameters in OVX mice induced by CSEs, acacetin, and linarin.

	Animals	Control Mice	OVX Mice	OVX Mice		
Parameters				20 mg/kg CSE	20 mg/kg Acacetin	20 mg/kg Linarin
Initial body weight (BW; g)		21.85 ± 0.34 ^a^	21.80 ± 0.29 ^a^	21.70 ± 0.30 ^a^	21.87 ± 0.19 ^a^	21.92 ± 0.28 ^a^
Final BW (g)		23.40 ± 0.36 ^c^	26.52 ± 0.29 ^a^	25.03 ± 0.45 ^b^	24.92 ± 0.31 ^b^	24.89 ± 0.42 ^b^
ADG (mg/d)		21.53 ± 0.64 ^c^	65.56 ± 0.54 ^a^	46.30 ± 3.87 ^b^	42.20 ± 1.80 ^b^	41.34 ± 2.40 ^b^
ADFI (g/d)		3.33 ± 0.06 ^a^	3.16 ± 0.10 ^a^	3.11 ± 0.06 ^a^	3.14 ± 0.06 ^a^	3.17 ± 0.06 ^a^
FER		0.47 ± 0.01 ^c^	4.37 ± 2.88 ^a^	1.08 ± 0.10 ^b^	1.00 ± 0.04 ^b^	0.94 ± 0.05 ^b^
LW		1.56 ± 0.05 ^a^	1.06 ± 0.07 ^b^	1.12 ± 0.04 ^b^	1.12 ± 0.03 ^b^	1.11 ± 0.04 ^b^
LW/BW(%)		6.66 ± 0.10 ^a^	4.04 ± 0.04 ^c^	4.49 ± 0.08 ^b^	4.51 ± 0.06 ^b^	4.47 ± 0.08 ^b^
GPT		22.25 ± 0.75 ^a^	22.29 ± 0.70 ^a^	21.50 ± 0.56 ^a^	21.33 ± 0.33 ^a^	21.67 ± 0.80 ^a^
GOP		49.25 ± 1.11 ^a^	49.29 ± 0.87 ^a^	50.00 ± 1.06 ^a^	49.80 ± 0.97 ^a^	49.50 ± 0.56 ^a^

Female C57BL/6 mice (11 weeks of age, 20-25 g) were surgically ovariectomized and orally supplemented with 20 mg/kg Cirsium setidens water extracts (CSEs), 20 mg/kg acacetin, and 20 mg/kg linarin daily for 8 weeks. Abbreviations: BW, body weight; ADG, average daily gain; ADFI, average daily food intake; FER, food efficiency ratio; OVX, ovariectomy; LW, liver weight; GPT, glutamic pyruvic transaminase; GOT, glutamic oxaloacetic transaminase. Values are means ± SEMs of 10 mice. Respective values in the same column not sharing the same superscript differ at *p* < 0.05.

**Table 3:** Effects of CSEs, acacetin, and linarin on blood lipid profile of OVX mice.

	Animals	Control Mice	OVX Mice	OVX Mice		
Parameters				20 mg/kg CSE	20 mg/kg Acacetin	20 mg/kg Linarin
TC		77.00 ± 0.91 ^d^	101.14 ± 1.65 ^a^	88.17 ± 2.09 ^c^	96.17 ± 0.95 ^b^	87.67 ± 5.58 ^c^
TG		54.86 ± 3.62 ^b,c^	75.25 ± 5.30 ^a^	62.67 ± 4.12 ^b^	57.60 ± 3.22 ^b,c^	53.17 ± 3.85 ^c^
HDL-C		41.71 ± 1.55 ^a^	31.00 ± 3.34 ^c^	36.67 ± 1.02 ^b^	44.50 ± 1.73 ^a^	39.33 ± 1.63 ^a,b^
LDL-C		30.95 ± 2.88 ^c^	58.46 ± 2.63 ^a^	38.97 ± 2.68 ^b^	40.15 ± 0.96 ^b^	37.70 ± 4.83 ^b^

C57BL/6 female mice (11 weeks of age, 20–25 g) were surgically ovariectomized and orally administrated with 20 mg/kg Cirsium setidens water extracts (CSEs), 20 mg/kg acacetin, and 20 mg/kg linarin daily for 8 weeks. Abbreviations: OVX, ovariectomy; TC, total **cholesterol**; TG, triglyceride; HDL-C, high-density lipoprotein cholesterol; LDL-C, low-density lipoprotein cholesterol. Respective values in the same column (mean ± SEM, *n* = 10) not sharing a small letter are different at *p* < 0.05.

## Data Availability

All the data presented in this study are included in the article.

## Abbreviations

BMD, bone mineral density; CAII, carbonic anhydrase II; ClC-7, chloride channel 7; CSEs, Cirsium setidens water extracts; CTX-1, carboxy-terminal telopeptide of type 1 collagen; MMP-9, matrix metalloproteinase-9; NTX-1, amino-terminal telopeptide of type 1 collagen; OPG, osteoprotegerin; OVX, ovariectomy; PICP, procollagen type 1 carboxy-terminal propeptide; PINP, procollagen type 1 amino-terminal propeptide; RANKL, receptor activator of nuclear factor-κB ligand; TNSALP, tissue non-specific alkaline phosphatase; TRAP, tartrate-resistant acid phosphate; V-ATPase, vacuolar-type H(+)- ATPase.
